# Cross-Temporal and Cross-National Poverty and Mortality Rates among Developed Countries

**DOI:** 10.1155/2013/915490

**Published:** 2013-06-09

**Authors:** Johan Fritzell, Olli Kangas, Jennie Bacchus Hertzman, Jenni Blomgren, Heikki Hiilamo

**Affiliations:** ^1^Centre for Health Equity Studies (CHESS), Stockholm University/Karolinska Institutet, 106 91 Stockholm, Sweden; ^2^The Social Insurance Institution of Finland (KELA), P.O. Box 450, 00101 Helsinki, Finland

## Abstract

A prime objective of welfare state activities is to take action to enhance population health and to decrease mortality risks. For several centuries, poverty has been seen as a key social risk factor in these respects. Consequently, the fight against poverty has historically been at the forefront of public health and social policy. The relationship between relative poverty rates and population health indicators is less self-evident, notwithstanding the obvious similarity to the debated topic of the relationship between population health and income inequality. In this study we undertake a comparative analysis of the relationship between relative poverty and mortality across 26 countries over time, with pooled cross-sectional time series analysis. We utilize data from the Luxembourg Income Study to construct age-specific poverty rates across countries and time covering the period from around 1980 to 2005, merged with data on age- and gender-specific mortality data from the Human Mortality Database. Our results suggest not only an impact of relative poverty but also clear differences by welfare regime that partly goes beyond the well-known differences in poverty rates between welfare regimes.

## 1. Introduction

Fighting poverty has always been at the centre of welfare state activities. There are several important reasons for such a focus, but a key issue is no doubt the relationship between poverty and ill-health and premature death, shown not least by several classical and historical investigations [1, 2]. 

The finding of the social gradient is also of interest when going from these historical studies to present discussions about poverty, inequality, and population health, as it indicates that not only the very poorest sections were hit but that relative poverty was also of importance. It is well known that countries with high absolute poverty rates today (e.g., World Bank indicators of 1 or 2 US dollars a day) also tend to be those with low life expectancy and high mortality risks. But what is the relationship between relative poverty rates and mortality risks among the richer countries of the world?

Assuming that the poorest people in rich countries do not live under absolute poverty, the relationship between variations in relative poverty rates and variations in mortality rates may seem less self-evident. However, this relationship has been at the centre of one of the most debated topics within the field of public health research and social epidemiology in recent decades, namely, the health impact of income inequality. It is actually one foundation of the so-called Wilkinson hypothesis, which basically states that it is not the level of affluence as such that matters among rich countries but rather how the pie of total economic resources is distributed [3]. This hypothesis is articulated in relation to the whole social structure, thus stating that it is income inequality as such, not only poverty, that kills. However, most evidence, on both the macrolevel of countries and the microlevel of individuals, suggests a curvilinear association between income and health, which implies that health gains can be made by transferring money from the richer to the poorer. If this is so, it means that not only income inequality but also—and even more evident—variations in poverty rates should be associated with population health. But can we evidence that cross-national variations in relative poverty rates are related to cross-national variations in survival possibilities in relatively rich countries?

In this study, we conduct a comparative analysis of the relationship between poverty and mortality across 26 developed countries over time. We utilize data from the Luxembourg Income Study [4] to construct age-related poverty rates across countries and time covering the period from around 1980 to 2005, merged with data on age- and gender-specific mortality data from the Human Mortality Database [5]. It is reasonable to assume that the consequences of poverty differ between gender and age groups. It is also a well-known fact that the causes of death vary by gender and age. For example, it has been shown that there were larger socioeconomic differences in men's mortality patterns than women's [6]. It follows that it seems to be a reasonable hypothesis that the poverty and mortality relationship could also be different between women and men. Therefore, it is important that we conduct sex- and age-disaggregated analyses. 

In the next section, we briefly present some of the arguments and empirical evidence of relevance to our study. Thereafter we present our data, methods, and analytical design. We then present our results, and the paper ends with a concluding discussion about our findings.

**Figure 1:**
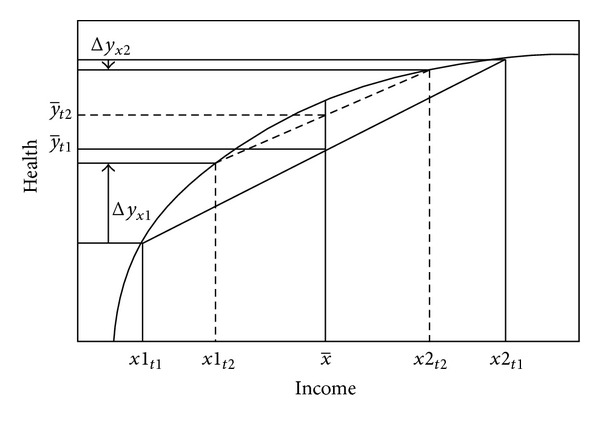
Theoretical connection between individual- and aggregate-level relationships between income and health From (Lundberg et al. [19], adapted from Rodgers [7]).

As mentioned, the idea that income inequality could influence population health was noted already in the typical curvilinear association of the so-called Rodgers curve. Partly based on empirical data, Rodgers [7] presented a model of how smaller income disparities and relative poverty at societal level are linked to better public health through differential impacts on individual health status among both low- and high-income earners. He argued that the health returns of income diminish at higher income levels, implying that this relationship is curvilinear [7, 8]. In the Rodgers example (Figure 1), the health of the low-income person *x*1 is much poorer than that of the high-income person *x*2 at *t*1. Redistributing income from *x*2 to *x*1 at *t*2 will result in an unchanged average income $\left(\overset{-}{x}\right)$, while average health $\left({\overset{-}{y}}_{t2}\right)$ improves. This is simply the result of the health gain among the poor (Δ*y*_*x*1_) being larger than the health loss among the rich (Δ*y*_*x*2_) as a consequence of this income redistribution. Rodgers also presented results from cross-national, cross-sectional analysis supporting the specification that countries with lower inequality had higher life expectancy.

Although Rodgers, and later Wilkinson [9], articulated how the whole income distribution could make a difference, it is evident from the hypothesis that what should particularly make a difference is how the relatively poor fare and how large a fraction of the population is at risk of poverty. 

The topic of income inequality and health has become a small research industry within social epidemiology, with some influences from economics and sociology, and numerous studies have been published, especially on the relationship between income inequality across American states and various health outcomes [10]. One major review [11] was largely in favour of the hypothesis, whereas another [12] was sceptical. A meta-analysis of multilevel studies linking income inequality to mortality and self-rated health lent support to the idea [13]. A recent global study investigating 140 countries also lends support to the hypothesis, but only in low- and middle-income countries [14].

In cross-national analyses, not least with regard to population health and poverty, it has become common procedure to group countries according to their specific mix of welfare production, that is, welfare regime [15, 16]. As noted by several authors [17, 18], the welfare modelling business has become a central part of welfare state research, starting with Esping-Andersen's famous trichotomy that he labelled on the basis of main political ideologies: the liberal, social-democratic, and conservative/corporatist regimes. The idea behind the regime approach goes beyond the welfare state in the stricter sense by looking at the nexus of the state, markets, and family. While Esping-Andersen identified three welfare state regimes among the countries he analysed, it has subsequently been common procedure to also include and identify additional clusters of Southern and Eastern European countries. Although our overall aim is to study the link between poverty and mortality, it is of obvious interest to note variations in this relationship by welfare state regime and to adjust our analyses by regime.

The role of welfare state programmes in population health has recently been highlighted. Not least within the NEWS project [19], initiated in collaboration with the WHO Commission on Social Determinants of Health, a number of studies were produced linking the specific design, generosity and coverage of social policy programmes to overall and age-specific mortality on the one hand, and to morbidity on the other [20–24]. These studies focused on the cash side of the welfare state and supported the idea that cash programmes of the welfare state have been of importance to public health during the second half of the 20th century. These studies did not investigate the role of welfare services, nor did they study any specific mechanisms behind the associations found. However, the ability of these programmes to alleviate poverty was often referred to as a key factor in cross-national variations in mortality rates. Of course, the programmes of the welfare state are likely to also influence other more proximal health-related factors that could influence mortality risks. In this study we will explore the relative poverty argument directly, by making use of the best sources for comparative studies on poverty and mortality over a 25-year period. We partly overcome the small-*N* problem that occurs in most cross-national studies by using multiple waves of data for each country included.

Although we will not examine the mechanisms, it is still necessary to briefly mention some of the possible multiple pathways linking relative poverty and mortality. Overall mortality has decreased in recent decades in developed countries (with Russia as the only exception). The question is whether the incidence of relative poverty has delayed or prevented a fall in mortality in the countries included in our analysis. The experience of living in relative poverty may be connected to unhealthy habits and continuous stress, as well as negative consequences more or less directly stemming from a lack of resources, for example, not being able to consume healthy food or live in adequate housing, or moving to a neighbourhood with more safety, better primary health care or better schools and other services. We believe many of these factors may work in a causal chain rather than as contradictory mechanisms [25]. In so far as psychosocial processes are at work, it seems more reasonable to assume that they have a material base than to regard the material and psychosocial as representing two opposite and mutually exclusive poles.

## 2. Material

Our two main data sources are the Luxembourg Income Study (LIS) and the Human Mortality Database (HMD). The LIS is a cross-national harmonized database that includes multiple waves of microdata for a number of countries. It has a focus on income inequality and poverty, but also includes a great deal of information on aspects such as family situation, and employment status. Wave 1 started around 1980 with approximate five-year intervals, so that Wave 6 of the data is from around 2005 (for a thorough presentation of the database see [26]). The LIS is commonly regarded as the best source of cross-national comparisons of poverty and income inequality. The HMD, maintained by the University of California, Berkeley, and the Max Planck Institute of Demographic Research, provides detailed open access mortality and population data for a number of countries for years reaching from the 1800s to around 2010. Currently, the HMD includes information for 37 countries, which are partly the same and partly different to those in the LIS database. 

In our study, we include all countries from the LIS that have at least two waves of data from the same original survey source, and for these countries, all LIS waves for which mortality data were also available in the HMD for corresponding years. This led to a country sample of 26 rich countries with two to six waves, a total of 122 data points (see Table 1). The LIS data was accessed and analysed during January-February 2011. Countries included are Australia, Austria, Belgium, Canada, the Czech Republic, Denmark, Finland, France, Germany, Hungary, Ireland, Israel, Italy, Luxembourg, the Netherlands, Norway, Poland, Russia, the Slovak Republic, Slovenia, Spain, Sweden, Switzerland, Taiwan, the United Kingdom, and the United States.

## 3. Analytical Approach, Methods, and Variables

We investigate four nested models in which the dependent variable is the logged mortality rate and the exposure variable is the poverty rate. Mortality rates were assessed for three gender-specific age classes: infants (aged < 1 year), children (aged 1–17 years), and adults (aged 25–64 years). Data on deaths and populations at risk were collected for one-year age bands for each country from the HMD for all LIS waves, and for three following years of each wave. While infant mortality rates were used as such, age-standardized mortality rates for the age groups 1–17 and 25–64 were calculated to adjust for the different age structures of the countries. In these calculations, we used the direct method and the European standard population [27]. The age-standardized rates thus represent what the crude rates would be if the populations of the countries had the same age distribution as the European standard population. The age-standardized mortality rates were assessed as deaths per 1,000 person years, over four-year periods (from each wave until three years later), to allow for exposure time. However, for infant mortality, we only took into account the immediate year. In the multivariate regressions, the calculated rates were logged in order to normalize the skewed mortality data.

Poverty rates were calculated using a standard income poverty head-count measurement in which individuals living in households with equivalent disposable income lower than a certain percentage of median income are regarded as poor. Accordingly, we measure income after taking into account welfare state transfers and taxes. In order to be able to compare households of different sizes, each household's disposable income is divided by the square root of the number of persons in the household. The proportion of poor households will of course be partly determined by where we set the threshold. Evidently, the nature of poverty in terms of both the size of income and, for the countries analysed here, its consequences will become more severe the further we move from the national median. In our analyses we have employed a more severe definition than the usual 60% threshold, setting the poverty threshold at 40% of the national median. Poverty rates in each country and each wave were calculated separately for children (aged < 18) and working-age adults (aged 25–65). With the data at hand, we cannot have a perfect age match between the poverty rates and the mortality rates. Thus, the total child poverty rates are used as the exposure for both our child mortality analyses, and there is also a one-year mismatch for the adults. The latter mismatch is highly unlikely to have any effect on our results.

As confounders we consider the following variables. The LIS wave number is included to allow for time-related changes in poverty and mortality rates. The wave number also is an indicator variable pertaining to the more or less automatic decline in mortality that takes place in all countries. GDP per capita/1,000 US Dollars was derived from Penn's world tables [28] that contain information on the GDP per capita levels for all the countries included in our analyses. The GDP levels are adjusted to changes in cost of living across time and space and are given in 2005 US dollars. Social spending figures are taken from OECD databases. The social spending measure includes both benefits in cash and in kind. Administrative costs are also included, but the inclusion of the costs for running the schemes is not a major problem as these costs comprise only 2–4% of all expenditure. A more nuanced way of studying the impact of welfare spending would have been to use disaggregate spending data, that is, to separate cash and in kind benefits used for children, elderly, health care, various income maintenance programs, and so forth. However, this kind of analysis falls outside the scope of this particular paper and is a task for future studies. Here we simply assume that the overall social spending level reflects the state's commitment to citizens' welfare. and are expressed as a percentage of GDP. Russia and Taiwan are not included in this database; therefore, data for these countries are derived from other sources [29, 30]. Because data for Russia and Taiwan are adapted from nonstandard OECD sources, they are not totally comparable. Therefore, we have run sensitivity tests with and without these countries. The omission of Taiwan did not change the results, while the exclusion of Russia had a strong impact. Our data set is unbalanced; that is, data are not available for all countries and all years; therefore, we also run control analyses for the balanced data. Whereas the omission of Russia had the strongest impact, the omission of other outliers was not highly significant (see further discussion below). We also include dummy variables for the *welfare state regime* each country belongs to (see Table 1). The classification follows the more or less standard classifications. The latter variables were added in the model one at time to better investigate their associations.

Our analytical approach is first to inspect bivariate plots to observe the general pattern of the relationship between age-specific mortality rates and the background variables. We start by looking at developmental pattern in mortality over cross-sections and welfare regimes. Thereafter, we proceed to multivariate analyses to observe how the bivariate relationships will change when other variables are included in the regression models. For regression analyses, we used pooled cross-sectional time-series methods. These methods take advantage of the panel structure of the data while taking care of the correlations of data points between waves using panel-corrected standard errors [31–33]. In these analyses, we use country as the panel variable and wave as the time variable. Although partially solving the small *N*-problems, the pooled cross-sectional time-series method results in problems of spatial and longitudinal autocorrelation and heterogeneity. There are a number of regression techniques available to deal with the special problems of analysing pooled data, each with its own weaknesses, and results seem to be highly sensitive to the specific method applied [34–38]. Pooled regressions were run using the STATA 12 cross-sectional time-series package using Prais-Winsten regressions. Here we tested two possible ways to model the autocorrelation: (1) the PSAR(1) model uses autoregressive (AR1) autocorrelation that is panel specifically calculated. The positive side is that it is tailored for each panel separately, and the negative side is that it may be unstable if there are few cross-sections; (2) the AR(1) model uses an autocorrelation structure that is common for all panels. In order to further test the robustness of our results, we separately ran both AR(1) and PSAR(1) models. In practice, the results were robust for the different methods applied, and although the standard errors varied the interpretations of the results did not.

## 4. Results and Discussion

### 4.1. Infant Mortality Rates

**Figure 2:**
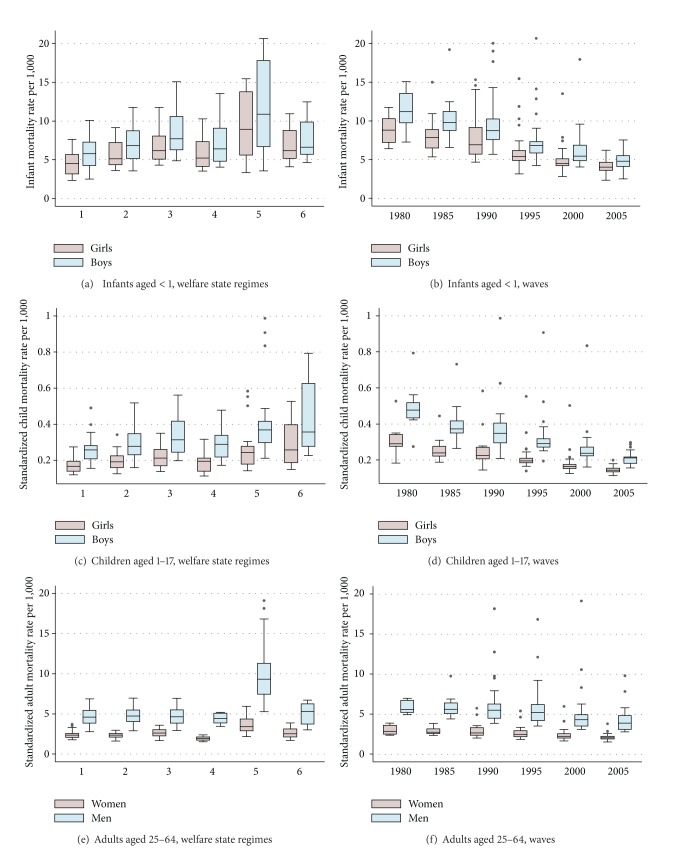
For infants (aged < 1), crude mortality rates per 1,000 and for children (aged 1–17) and adults (aged 25–64), standardized mortality rates per 1,000 among females and males in different welfare state regimes (1 = Nordic; 2 = Central European; 3 = Liberal; 4 = Southern European; 5 = Postsocialist; 6 = Other) and across waves, 1980 to 2005.

Figures 2(a)–2(f) show the magnitude and variability of infant, child, and adult standardized mortality rates by sex in different welfare state regimes and over time. We begin by looking more closely at infant mortality rates (Figures 2(a) and 2(b)). In the regression models to be shown later we will use logged mortality for girls and boys together, but in these more descriptive figures we show the raw figures for each sex. The box-plots [39] display medians and distributions of country-based and wave-based observations around the median values. 

The first observation from the box-plot figure is that infant mortality rates among boys are higher than among girls (7.99 per 1,000 boys and 6.43 per 1,000 girls, on average across countries and time points). While this gender gap holds for all welfare regimes there is regime- and country-based variation in the width of the gap, widest in the Postsocialist countries. As can be seen, the mortality rates are the lowest in the Nordic group (on average 4.56 for girls and 5.92 for boys) and there is relatively small variation between the four countries included in this cluster. The starting levels are already low, and there is a modest absolute decline in infant mortality. On the other end of the continuum, we find the former socialist countries to have both the highest mortality rates and the highest variation between nations, but also the highest absolute decline in infant mortality. There is substantial variation over time as well, and as evident in Figure 2(b), there is a downward trend over the waves concerning both levels and cross-national variation.

**Figure 3:**
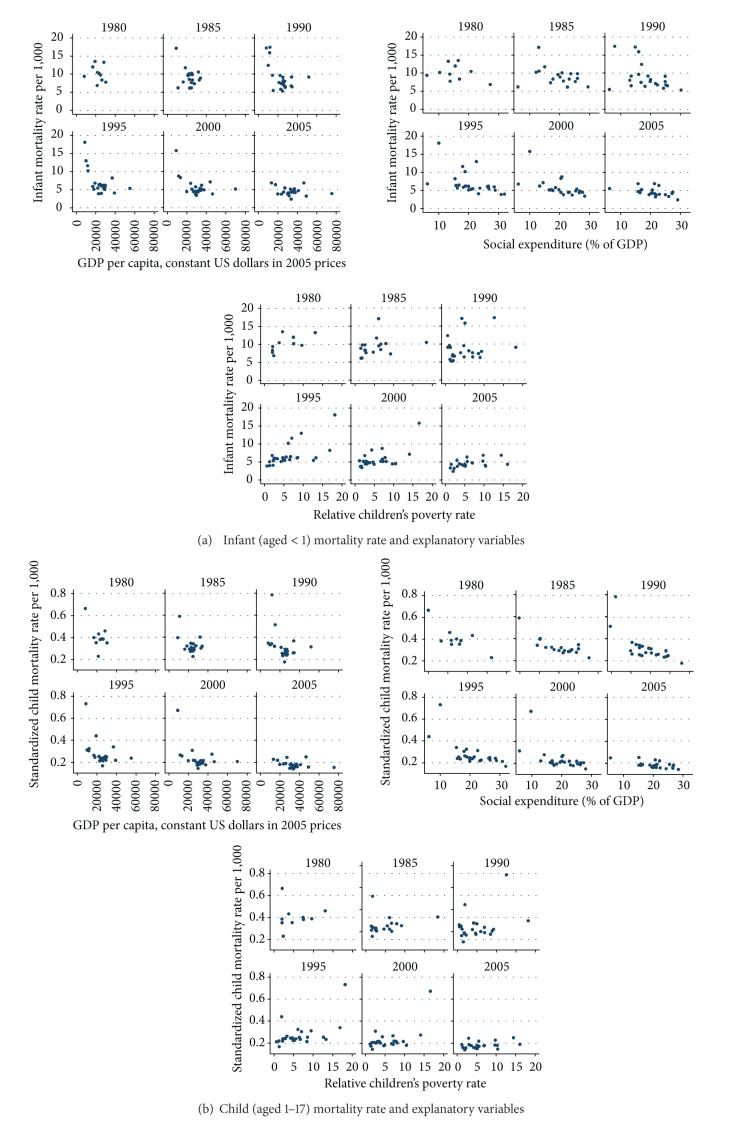

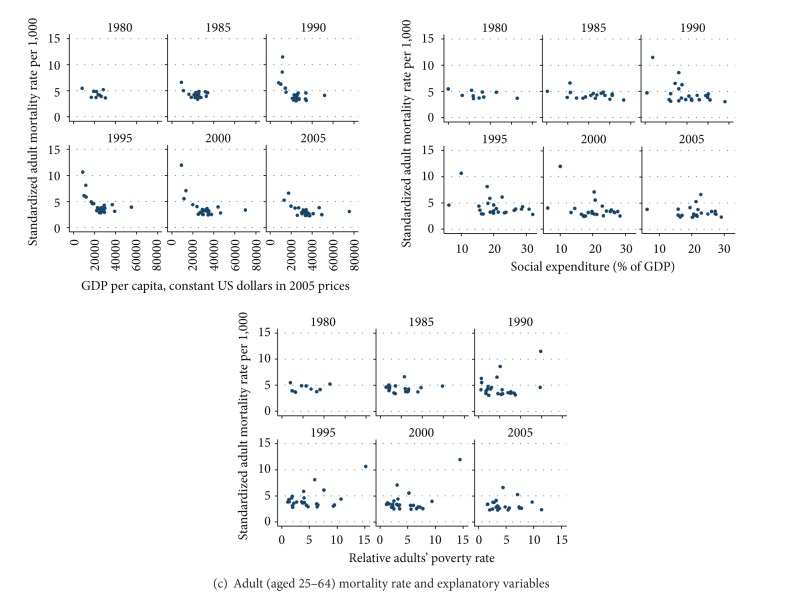
Relationships between explanatory variables (GDP per capita, social spending, and relative poverty rates) and infant crude mortality rate and child and adult standardized mortality rates, 1980–2005.

Bivariate scatterplots between infant mortality rates and the background variables we will later use in our multivariate models are shown in Figure 3(a). In pooled data, that is, where all cross-sections are merged into one, the relationships between predictors and infant mortality rates are in the expected direction but not always convincingly high. The overall correlation in the pooled data is the highest between infant mortality rate and GDP, indicating that infant mortality is conditioned by the wealth of the nation and all the factors linked to GDP. However, GDP is not only an indicator of economic prosperity but also represents a more general modernization trend that includes better food, better health care, better sanitation, access to clean water, and so forth—factors regarded as important in combatting infant mortality. In line with earlier research, we can also note the curvilinearity in the association between economic prosperity and infant mortality.

The second strongest correlation in the pooled data is the one between mortality and social spending, representing the magnitude of the public commitment to the social protection of the populace. Here as well the pattern is rather constant over cross-sections (Figure 3(a)): the larger the share of GDP that is made up of social spending, the lower the infant mortality rate. The association tends to be stronger in later periods of observation.

Contrary to our initial expectations, the link between infant mortality and child poverty rates is also relatively low in the pooled data. Whereas relative poverty is rather strongly correlated with mortality in the first and last waves, the correlations in Waves 2 and 3 are rather weak. Our interim conclusion is that the level of prosperity of the country and the magnitude of the welfare state matter, and that the impact of the welfare state is mirrored in lower levels of child poverty and inequality, which in turn partially combat new-born deaths.

An intriguing question is to what extent, if any, these bivariate relationships are robust when they are analysed simultaneously. In Table 2, we present results from regression analyses in which we step-wise include additional variables such as trend (wave), GDP per capita (1,000 US dollars in 2005 values), social spending and, finally, the welfare state regimes as dummies. In the last model (4) the Nordic welfare regime is used as a reference and is left out of the models. 

We ran the models separately for infant girls and boys, because the results turned out to be very similar we show them for both sexes combined. However, detailed results of the gender-specific analyses can be found in Tables 5(a) and 5(b). In the first model of Table 2, including only the poverty rate and the wave variable, the coefficient for poverty is significant. The coefficient of the association between poverty and logged mortality rate from this model can be statistically interpreted as follows: a one percentage-point increase in child poverty corresponds to about a 2% increase in infant mortality. The introduction of GDP per capita/1,000 US dollars (Model 2) does not change the picture. The inclusion of social spending (Model 3), as expected, leads to an attenuation of the poverty estimate—by about 40%. The statistical explanation for the strong attenuation of the poverty estimate when social spending is added is the strong association between social spending and poverty rates. So it seems that the welfare state matters for relative poverty, and relative poverty matters for infant mortality. 

Finally, when welfare regimes are introduced (Model 4), the poverty estimate remains about the same. The welfare regimes obviously capture not only different welfare state characteristics but also different levels of economic prosperity, since the coefficient for GDP totally disappears. In Model 4, controlling for poverty, wave, GDP, social spending and welfare regime, infant mortality rates are significantly higher in Central European, liberal, and especially Postsocialist regime types compared to the benchmarking Nordic regime (the reference category), while the Southern European and “other” regimes do not significantly deviate from the Nordic one. These regime differences are notable, especially if one bears in mind that they are not captured by differences in poverty, economic prosperity, or social spending. This evident variation between the regime types highlights that the causes of differences in population health statistics are multifactorial, and we are not able to fully capture this with the variables in our regression models. 

### 4.2. Mortality Rates among Children 1 to 17 Years of Age

When we move from the new-borns to older children, the risk of death radically diminishes. This is also reflected in the age-standardized mortality rates in the age group 1 to 17. Figures 2(c) and 2(d) give the variability in these rates by welfare state regime type and across waves. Again, there is an overrepresentation of boys in the death toll (the average age-standardized mortality rate over countries and time points is 0.22 per 1,000 girls and 0.33 per 1,000 boys aged 1–17). In the Postsocialist regime, the average death rate (0.27 among girls, 0.42 among boys) is about 1.5-fold compared to the low Nordic numbers (0.17 among girls, 0.26 among boys). In this age group, the country group “other” stands out with relatively high mortality rates but also very large variation. As seen in Figure 2(d), there is a clear trend here as well towards lower death rates in time (in average, from 0.31 to 0.15 among girls and from 0.49 to 0.21 among boys). The fact that we have an unbalanced panel can of course influence the magnitude of this downward trend, but the overall trend is general within all countries.

Figure 3(b) shows the crude relations between the pooled data of the age-standardized death rates and the three main explanatory factors. Especially social spending has a relatively strong association with child mortality, whereas the associations of GDP and relative child poverty with mortality are more modest. 

In order to cross-check the extent to which the results are biased by the former communist countries, we ran controls in which the countries in the Postsocialist cluster were excluded. In general, correlations between GDP and mortality became weaker, but the signs were not changed. Correlations between social spending and mortality became stronger, however, and correlations between poverty and mortality became stronger or remained almost the same. 

In Table 3 we show the results of our pooled cross-sectional time series analysis for this age group for girls and boys together, because the results of gender-specific analyses proved to be almost identical in terms of regression coefficients (see Tables 6(a) and 6(b)). The analytical strategy is basically the same as for infants, although the logged age-standardized mortality rates are now calculated as the average of the LIS years' mortality plus the following three years' mortality to allow for exposure time on mortality after our poverty measurements. The basic story for the age group 1 to 17 is also very much the same as what we showed for the infants (Table 2). The poverty estimates, especially in the two first models, and estimates for most of the other variables have similar magnitude as in the case of infant mortality. An important exception to this is the welfare regime. In terms of mortality among children aged 1–17, the liberal and Southern European regimes fare significantly better compared to the Nordic regime after the other covariates are adjusted for. Other regimes do not differ significantly from the Nordic one. At the same time, we can note that the poverty estimate actually doubles when welfare regime is adjusted for (compare Models 3 and 4). We will return to this finding in our final discussion. 

### 4.3. Mortality Rates among Adult Men and Women

When comparing adult and mortality rates with child mortality rates, interesting shifts in the rank order of “good” and “bad” regimes can be observed. Whereas in both groups of children (Figures 2(a)–2(d)) the Nordic welfare cluster displayed the lowest mortality rates, among adults the Southern European cluster outperforms the Nordic one (Figure 2(e)). The figure reveals the exceptionally high mortality rates among males in the Postsocialist countries, where the average age-standardized mortality rate across time points is as high as 10.06 for men and 3.69 for women; the corresponding figures for the Southern European cluster are 4.43 and 1.93. As in the case of child mortality, there is a general downward trend over time (Figure 2(f)).  Figure 3(c) once again displays the well-known curvilinear relationship between mortality and GDP per capita; the mirror picture of this is the relationship between GDP and life expectancy. Neither social spending nor relative adult poverty rate displays any clear-cut relationship with mortality. Although the relationship is somewhat different in different waves, the general message is that the bivariate plots basically show no association. 

Results from pooled cross-sectional regressions are shown separately for women (Table 4(a)) and men (Table 4(b)). In general, the association between poverty and mortality is weaker in the models for the adult population than for children. Starting with the results for women, we can note that poverty remains significantly and positively associated with mortality across all four models. The general picture that the poverty estimate attenuates when social spending is controlled for is also evident for women in the same manner as we saw earlier for infants and children. Somewhat oddly, we find that the poverty estimate actually increases when welfare regime type is also adjusted for (compare Model 4 to Model 3). Scrutinizing the estimate for regime, we note that the association between regime and adult mortality is different to that between regime and infant mortality (Table 2), but somewhat similar to that between regime and child mortality (Table 3). Compared to the Nordic regime, the Central and especially Southern European regime types show statistically significantly lower mortality rates, whereas the Postsocialist regime shows higher mortality rates among women when poverty, wave, GDP, and social spending are controlled for. 

Turning to the results for men (Table 4(b)), the picture is somewhat less clear. Although the poverty estimates as such are not lower than for women, the variability, as evident in the high *P*-values, is much higher. And, somewhat strangely, the poverty estimate has its largest value and is clearly significant only in the final model. Again, we find a different order across the regime types. The Postsocialist cluster has an extremely high estimate, especially considering all the other covariates we have adjusted for. Apart from this cluster we can note that, in comparison to the Nordic regime, the Southern European, liberal, and “other” regimes have lower adjusted male mortality rates. 

This difference between Southern and Northern Europe has also been corroborated by other cross-national research on mortality differences [40]. But here it seems as if these differences, for both women and men, are accentuated by the fact that we control for the other welfare state-related variables. Simultaneously, this also accentuates the effects of poverty for both women and men. 

### 4.4. Sensitivity Analyses

We have performed a number of sensitivity analyses with regard to the inclusion/exclusion of countries and setting a higher poverty threshold. We also tested the impact of income inequality (as expressed by the Gini coefficient). We argue that the 40% poverty threshold comes closer to the “absolute” poverty level, not least combined with the national wealth indicator (GDP), than the 60% poverty threshold, which comes closer to income inequality measured using the Gini coefficient. However, as Gini and poverty measures are strongly correlated they cannot be used simultaneously as explanatory variables.

The correlation between the whole population-level Gini and relative poverty rate with 40% threshold is 0.85, and Gini and relative poverty rate with 60% threshold is 0.89.

When it comes to deviant cases we have one country that stands out: Russia. During the time span covered by this study, Russia had high poverty rates and extreme death risks, especially visible among adult males. Therefore, when we reran all regressions omitting Russia, the estimates changed substantially. By and large our poverty estimates attenuated by about a third for infants, the degree of attenuation varied across the models for children aged 1–17, and among adults the poverty estimate became insignificant.

### 4.5. Methodological Considerations

We chose to study the possible influence of poverty with level rather than change. This choice was made mainly for theoretical reasons, as we suggest that it is the long-term and broad difference in poverty that matters rather than any yearly fluctuations [36, 41]. Thus, we assume that it is the magnitude of poverty that is lethal. We realize that models focusing on change would capture unmeasured heterogeneity but, on the other hand, such models also increase the noise-to-signal ratio. In the end, in line with Babones [42] in his comparative analysis of income inequality and health, we note that a major complication for any fixed effects model is the remarkable stability for both variables over time. However, it should be noted that some of the fixed effects are still included through the dummies for the LIS wave and the welfare state regimes. 

Another methodological concern in our study is the fact that we have an unbalanced panel structure. In other words, we have different countries in different waves. Although statistically speaking our method takes this into account, it may still have an influence on our findings. This is an analogy to the finding from a simulation analysis by Pop et al. [14] suggesting that the composition of the sample of high-income countries may be crucial. Still, in sensitivity analyses we found that a balanced panel gave largely similar results. In summary, it seems that the relative poverty rates are of importance to child mortality for the sample of countries and the period examined. This is also in line with earlier research showing a stronger association between income inequality and infant and child mortality than between income inequality and adult mortality [43]. Further, Galobardes et al. [44] found evidence of socioeconomic resources in childhood and later in life having both direct and indirect effects on mortality patterns. Primarily two models have been suggested in life course epidemiology: accumulation and critical periods during life [45]. Poor socioeconomic resources in childhood are associated with morbidity patterns in adulthood, particularly diseases such as stomach cancer and hemorrhagic stroke. The magnitudes of deprivation among children and the effect of the association vary between countries and have also been shown to be influenced by the design of the welfare state, such as choices of social policy/redistribution.

## 5. Conclusions 

The aim of this study was to analyse the effect of relative poverty upon mortality rates among three age groups—infants, children, and working-age adults, also stratified by gender. We used a low threshold (40% of median) to measure relative poverty, which thereby measures more severe poverty prevalence. Our time period is 1980–2005, and we have an unbalanced time series for 26 countries belonging to the rich world but also including Postsocialist countries from Eastern Europe. Our method is pooled cross-sectional time series analysis. We have recently seen a number of studies that go beyond the cross-sectional picture between income inequality and mortality [14, 42, 46, 47]. To our knowledge, this is one of the first studies to go beyond the cross-sectional picture with a focus on poverty rather than inequality. There is ample evidence of profound differences in poverty across welfare regimes [48–50], suggesting that poverty, welfare regime, and mortality also may be interrelated. 

Our results are basically the following: we find support for the assumption that the prevalence of poverty is of importance. The strength and level of significance vary depending on which additional variables are included in the model. When social spending is included, the poverty estimate for children attenuates by a third. A statistical explanation for this is the strong and robust association between poverty and social spending. When thinking about the order of impacts, one can, with overwhelming empirical support, argue that social spending is causally related to poverty: the higher the spending level in a country, the lower the poverty levels. Welfare state matters for poverty, and poverty matters for child mortality. We also include welfare regime type in our final models. We anticipated that if we took into consideration the welfare regimes' belongingness, the relative role of poverty rates would be “eaten up.” However, for children the effect on the poverty estimate was negligible, and for adults the inclusion of welfare regime fortified the connection between poverty and mortality. 

This does not influence the poverty and mortality association for children, but it is important to note that the regime type as such has a clear influence on child mortality, even when controlling for GDP and social spending. In other words, this result suggests that there are other regime-specific factors that are important.

For adults, the results are less straightforward. Here the results depend on which model you focus upon. Interestingly enough, for both women and men we find that the poverty estimate becomes stronger when welfare state regime type is also controlled for. The reason for this is not self-evident, but from earlier research we know that several of the Southern European countries are ranked at the top of life expectancy figures in Europe and worldwide. We also know that they are less favourably ranked when it comes to poverty rates. In a sense, the regime variable captures whatever it is that is specifically health-promoting in these countries, and the resulting poverty estimate is thereby adjusted for that regime-specific aspect. When making such an adjustment, the remaining effect of poverty increases substantially. 

Another intriguing result, then, is that welfare regimes do not treat all age groups similarly. When it comes to the Nordic welfare model, it seems to be good for infants and children but is no longer superior in older age groups, and some Central and Southern European countries outperform it. The results also show exceptionally high mortality rates among males living in the Postsocialist countries. This result, in turn, indicates that welfare state and poverty have an impact on mortality, but there are other factors in play, such as drinking and eating habits and the way healthy and unhealthy behaviour is distributed between socioeconomic groups, according to income and education attainment levels.

Our study is definitely not the final answer to the question of whether or not the prevalence of poverty in relatively rich countries still has an influence on death risks. Our study is somewhat different to most of the cross-country studies linking poverty and mortality. They have used either a more worldwide inclusion of countries (but the question then becomes somewhat different) or a much smaller sample of countries and have particularly been totally cross-sectional in their design. Moreover, we have used age-specific analysis when it comes to both poverty calculations and mortality rates, thereby further specifying the tests.

Finally, as our study is based on large-scale macrophenomena we obviously have several mediating factors. However, a policy recommendation from our study is that national governments invest in eliminating child poverty. This is likely to have positive population health effects from both a short- and long-term perspective. From cross-national poverty analyses we know that universal, redistributive social policies are key instruments in reducing poverty [49]; if such policies are also coupled with social investment policies for young children, such as high-quality day care, this not only reduces poverty here and now but is also likely to be a good investment for the future. 

## Figures and Tables

**Table 1:** Countries and LIS waves included in the analysis, grouped by welfare state regime.

Country	LIS Waves					
	1	2	3	4	5	6
Nordic model						
Denmark		1987	1992	1995	2000	2004
Finland		1987	1991	1995	2000	2004
Norway	1979	1986	1991	1995	2000	2004
Sweden	1981	1987	1992	1995	2000	2005
Central European model						
Austria		1987		1994	2000	2004
Belgium		1985	1988	1995	2000	
France	1979	1984	1989	1994	2000	
Germany			1989	1994	2000	2004
Luxembourg		1985	1991	1994	2000	2004
Netherlands		1987	1991	1994	1999	2004
Switzerland	1982		1992		2000	2004
Liberal model						
Australia	1981	1985	1989	1995	2001	2003
Canada	1981	1987	1991	1994	2000	2004
Ireland		1987		1995	2000	2004
United Kingdom	1979	1986	1991	1995	1999	2004
United States	1979	1986	1991	1994	2000	2004
Southern European model						
Italy		1986	1991	1995	2000	2004
Spain	1980		1990	1995	2000	2004
Postsocialist model						
Czech Republic			1992	1996		2004
Hungary			1991	1994	1999	2005
Poland		1986	1992	1995	1999	2004
Russia			1992	1995	2000	
Slovak Republic			1992	1996		
Slovenia				1997	1999	2004
Other						
Israel		1986	1992	1997	2001	2005
Taiwan	1981	1986	1991	1995	2000	2005
Number of countries per wave	10	18	23	25	24	22

**Table 2:** Associations between logged infant (aged < 1) mortality rates and explanatory factors. Results from pooled cross-sectional time series analyses. *N* (countries): 26, *N* (observations): 122.

	Model 1		Model 2		Model 3		Model 4	
	Coef.	*P* values	Coef.	*P* values	Coef.	*P* values	Coef.	*P* values
Child poverty (40%)	0.020	0.011	0.022	0.001	0.013	0.080	0.015	0.088
Wave	−0.183	0.000	−0.155	0.000	−0.143	0.000	−0.179	0.000
GDP/1,000 US dollars			−0.010	0.001	−0.009	0.002	−0.001	0.707
Social spending					−0.019	0.000	−0.013	0.068
Welfare regime:								
Central European							0.216	0.000
Liberal							0.176	0.015
Southern European							0.115	0.272
Postsocialist							0.483	0.004
Other							0.135	0.188
Constant	2.478	0.000	2.626	0.000	2.980	0.000	2.555	0.000

**Table 3:** Associations between logged age-standardized child (aged 1–17) mortality rates and explanatory factors. Results from pooled cross-sectional time series analyses. *N* (countries): 26, *N* (observations): 122.

	Model 1		Model 2		Model 3		Model 4	
	Coef.	*P* values	Coef.	*P* values	Coef.	*P* values	Coef.	*P* values
Child poverty (40%)	0.021	0.001	0.020	0.000	0.009	0.025	0.018	0.004
Wave	−0.158	0.000	−0.135	0.000	−0.111	0.000	−0.124	0.000
GDP/1,000 US dollars			−0.009	0.000	−0.009	0.000	−0.005	0.017
Social spending					−0.023	0.000	−0.026	0.000
Welfare regime:								
Central European							−0.019	0.626
Liberal							−0.167	0.003
Southern European							−0.150	0.032
Postsocialist							0.051	0.485
Other							−0.138	0.158
Constant	−0.890	0.000	−0.725	0.000	−0.312	0.000	−0.325	0.000

**Table:** (a) Women 25–64

	Model 1		Model 2		Model 3		Model 4	
	Coef.	*P* values	Coef.	*P* values	Coef.	*P* values	Coef.	*P* values
Adult poverty (40%)	0.016	0.018	0.010	0.004	0.006	0.006	0.017	0.004
Wave	−0.077	0.000	−0.042	0.000	−0.034	0.000	−0.060	0.000
GDP/1,000 US dollars			−0.012	0.000	−0.012	0.000	−0.007	0.001
Social spending					−0.009	0.000	−0.006	0.009
Welfare regime:								
Central European							−0.051	0.041
Liberal							0.009	0.852
Southern European							−0.274	0.000
Postsocialist							0.232	0.004
Other							−0.125	0.244
Constant	1.150	0.000	1.376	0.000	1.518	0.000	1.359	0.000

**Table:** (b) Men 25–64

	Model 1		Model 2		Model 3		Model 4	
	Coef.	*P* values	Coef.	*P* values	Coef.	*P* values	Coef.	*P* values
Adult poverty (40%)	0.021	0.111	0.013	0.082	0.008	0.197	0.029	0.000
Wave	−0.091	0.000	−0.031	0.002	−0.024	0.042	−0.069	0.000
GDP/1,000 US dollars			−0.021	0.000	−0.021	0.000	−0.011	0.000
Social spending					−0.009	0.050	−0.007	0.001
Welfare regime:								
Central European							−0.058	0.070
Liberal							−0.195	0.001
Southern European							−0.256	0.000
Postsocialist							0.397	0.000
Other							−0.315	0.003
Constant	1.919	0.000	2.296	0.000	2.457	0.000	2.226	0.000

**Table:** (a) Girls < 1

	Model 1		Model 2		Model 3		Model 4	
	Coef.	*P* values	Coef.	*P* values	Coef.	*P* values	Coef.	*P* values
Child poverty (40%)	0.020	0.022	0.021	0.001	0.013	0.076	0.014	0.104
Wave	−0.176	0.000	−0.148	0.000	−0.136	0.000	−0.182	0.000
GDP/1,000 US dollars			−0.010	0.003	−0.009	0.007	0.002	0.681
Social spending					−0.019	0.000	−0.009	0.247
Welfare regime:								
Central European							0.236	0.000
Liberal							0.225	0.002
Southern European							0.180	0.067
Postsocialist							0.581	0.000
Other							0.295	0.007
Constant	2.328	0.000	2.493	0.000	2.828	0.000	2.257	0.000

**Table:** (b) Boys < 1

	Model 1		Model 2		Model 3		Model 4	
	Coef.	*P* values	Coef.	*P* values	Coef.	*P* values	Coef.	*P* values
Child poverty (40%)	0.023	0.002	0.022	0.000	0.014	0.066	0.016	0.054
Wave	−0.192	0.000	−0.164	0.000	−0.151	0.000	−0.172	0.000
GDP/1,000 US dollars			−0.009	0.001	−0.008	0.001	−0.004	0.064
Social spending					−0.019	0.000	−0.016	0.016
Welfare regime:								
Central European							0.194	0.000
Liberal							0.131	0.088
Southern European							0.049	0.674
Postsocialist							0.380	0.025
Other							−0.009	0.927
Constant	2.583	0.000	2.732	0.000	3.088	0.000	2.814	0.000

**Table:** (a) Girls 1–17

	Model 1		Model 2		Model 3		Model 4	
	Coef.	*P* values	Coef.	*P* values	Coef.	*P* values	Coef.	*P* values
Child poverty (40%)	0.021	0.001	0.019	0.000	0.008	0.020	0.016	0.008
Wave	−0.142	0.000	−0.123	0.000	−0.099	0.000	−0.112	0.000
GDP/1,000 US dollars			−0.008	0.000	−0.007	0.000	−0.004	0.004
Social spending					−0.023	0.000	−0.024	0.000
Welfare regime:								
Central European							0.009	0.823
Liberal							−0.121	0.047
Southern European							−0.130	0.061
Postsocialist							0.074	0.229
Other							−0.051	0.616
Constant	−1.194	0.000	−1.038	0.000	−0.623	0.000	−0.673	0.000

**Table:** (b) Boys 1–17

	Model 1		Model 2		Model 3		Model 4	
	Coef.	*P* values	Coef.	*P* values	Coef.	*P* values	Coef.	*P* values
Child poverty (40%)	0.021	0.001	0.020	0.000	0.009	0.044	0.018	0.004
Wave	−0.169	0.000	−0.141	0.000	−0.117	0.000	−0.136	0.000
GDP/1,000 US dollars			−0.009	0.000	−0.009	0.000	−0.004	0.160
Social spending					−0.024	0.000	−0.025	0.000
Welfare regime:								
Central European							−0.019	0.627
Liberal							−0.177	0.001
Southern European							−0.144	0.037
Post-socialist							0.084	0.267
Other							−0.153	0.070
Constant	−0.666	0.000	−0.507	0.000	−0.088	0.152	−0.139	0.105
